# Hydrothermal Synthesis and Optical Properties of Cr-Doped Bi_2_Se_3_ Topological Insulator Nanoplatelets

**DOI:** 10.1021/acsomega.6c02860

**Published:** 2026-06-03

**Authors:** Blaž Belec, Mattia Fanetti, Thanveer Thajudheen, Sandra Gardonio, Matjaz Valant

**Affiliations:** Materials Research Laboratory, University of Nova Gorica, 5000 Nova Gorica, Slovenia

## Abstract

Incorporation of magnetic Cr dopants into the Bi_2_Se_3_ topological insulator (TI) system has predominantly been achieved in bulk single crystals and thin films, whereas Cr doping at the nanoparticle level has not been realized. Cr-doped Bi_2_Se_3_ thin nanoplatelets were successfully synthesized via a hydrothermal route, establishing a wet-chemical approach to producing magnetically doped TI nanoparticles. Structural and quantitative energy dispersive X-ray spectroscopy compositional analyses reveal substitutional incorporation of Cr^3+^ into the Bi_2_Se_3_ lattice up to 4.8 ± 0.4 at. %, independent of the nominal precursor concentration, thereby defining the solid solubility limit of Cr in Bi_2_Se_3_ nanoparticles prepared under hydrothermal conditions. Excess Cr is accommodated through the formation of a Cr-rich secondary phase. UV–vis absorption spectroscopy demonstrates enhanced optical absorption across the 200–1000 nm spectral range upon Cr incorporation. In contrast, cathodoluminescence (CL) spectroscopy on individual Cr-doped nanoplatelets shows that despite Cr doping, the CL emission remains localized at nanoplatelet edges and exhibits spectral characteristics identical to those of pristine Bi_2_Se_3_. These results demonstrate that hydrothermal synthesis is a viable route for preparing Cr-doped Bi_2_Se_3_ nanoplatelets and provide insights into dopant incorporation limits and their impact on nanoscale optical properties.

## Graphical abstract

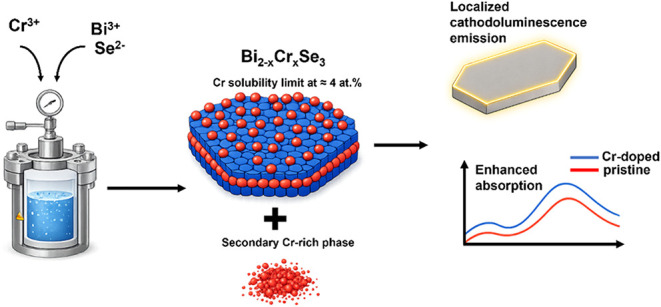

## Introduction

Magnetic doping of topological insulators (TIs) with 3d transition metal ions such as Cr^3+^ provides an effective route to tailor their electronic structure and explore the resulting properties.^1–3^ TI materials, with Bi_2_Se_3_ as a prototypical representative, are characterized by an insulating bulk and gapless metallic topological surface states (TSSs) located at their surfaces or edges.^4,5^ While these surface states are robust against nonmagnetic disorder, the incorporation of magnetic impurities breaks time reversal symmetry, modifies the band structure, and can open a gap at the Dirac point. Interplay between magnetism and topology can result in new physical phenomena, such as the quantum anomalous Hall effect, which make TI relevant for spintronics and quantum computing applications.^1–3,6–11^ Among the possible magnetic dopants, Cr^3+^ is particularly suitable because its ionic radius and valence closely match those of Bi^3+^, enabling charge-neutral substitution with minimal lattice distortion and thus avoiding resulting effects that could mask the intrinsic magnetic–topological interplay effects.

However, systematic investigation of magnetic doping effects in TIs requires high-quality materials, making synthesis methods that enable precise compositional control an active area of research.^3^ To date, Cr-doped Bi_2_Se_3_ with the composition Bi_2–*x*_Cr*_x_* Se_3_ has been prepared mainly as single crystals^12,13^ and thin films^7,14–20^ for studies of its fundamental electronic and transport properties. For single crystals grown by the Bridgman method, Cr incorporation is strongly limited by its low solubility in the Bi_2_Se_3_ lattice, which does not exceed 0.4 at. % at ≈600 °C.^12^ Besides low Cr solubility, a problem with this approach is the formation of a secondary Bi_2_Cr_4_Se_9_ phase which, when starting with low nominal Cr concentrations (0.1–0.8 at.%), grows as microcrystallites within the matrix. This can be overcome by starting with higher nominal Cr concentrations (1–3 at. %) and optimized growth conditions, particularly controlled cooling.^13^ This strategy enabled the growth of larger secondary phase crystals that could be separated from the highly diluted Cr-doped Bi_2_Se_3_ matrix, which contained only about 0.15 at. % Cr. In contrast, molecular beam epitaxy (MBE) has enabled the preparation of Cr-doped Bi_2_Se_3_ thin films with reported Cr concentrations ranging from 0.8 to 4.6 at. %,^7,14–20^ without detected secondary phases or Cr segregation. However, in many studies, the reported Cr concentration is derived from nominal growth conditions aimed to achieve distinct targeted composition and not determined with additional independent compositional verification of the actual dopant content.^15,16,18–20^

The study of the electronic properties of Bi_2_Se_3_ doped with Cr, synthesized by the Bridgman method or by the MBE, highlights the presence of Cr impurity states as the source of the observed modification of the Dirac cone in the material.^16,21^ Furthermore, investigation of the dynamic processes of photoexcited carriers in Bi_2_Se_3_ with varying Cr doping concentrations found that as the Cr doping concentration increases, the lifetime of the excited electrons decreases.^18^ This mechanism is attributed to relaxation through the impurity band induced by Cr doping, which provides additional recombination paths. In light of this, Cr doping may serve as a tool for tuning the carrier lifetime in Bi_2_Se_3_, potentially promoting its application in the field of optoelectronics. In optoelectronics, Bi_2_Se_3_ and more generally TIs are particularly interesting in their two-dimensional forms, such as nanoplatelets.^22^

At the nanoscale, properties arising from their unique electronic structure can be enhanced or new properties can emerge (e.g., optical), making TI nanoparticles promising for photonic and optoelectronic applications, especially in the UV–vis range, such as light concentrators in data storage devices,^23,24^ plasmonic devices,^23–29^ and biomedical diagnostics and therapy.^30–35^ Despite significant interest in Bi_2_Se_3_ nanostructures, particularly 2D nanoplatelets, and the optoelectronic potential of Cr-doped Bi_2_Se_3_, the preparation of Cr-doped Bi_2_Se_3_ via wet-chemical routes such as hydrothermal synthesis, which enables nanoparticle formation, has not yet been explored.

Here, we demonstrate for the first time the hydrothermal synthesis of Cr-doped Bi_2_Se_3_ thin nanoplatelets adopting a wet-chemical procedure that we developed for pristine Bi_2_Se_3_ nanoparticles.^36^ Structural and quantitative compositional analyses reveal a two-phase system composed of Cr-doped Bi_2_Se_3_ nanoplatelets with a Cr dopant concentration of 4.8 ± 0.4 at. %, determined using STEM-EDXS, which represents a solid solubility limit of Cr in Bi_2_Se_3_ nanoplatelets, together with a Cr-rich secondary phase formed from excess dopant. We also investigated the effect of dopant incorporation on the optical properties, finding that Cr^3+^ doping enhances absolute absorption in the UV–vis region, while preserving the characteristic edge-localized cathodoluminescence emission of Bi_2_Se_3_ nanoplatelets.^37^

## Methods and Materials

### Chemicals

Bismuth (III+) nitrate pentahydrate (Bi­(NO_3_)_3_·5H_2_O), Cr (III) nitrate nonahydrate (Cr­(NO_3_)_3_·9H_2_O selenium powder (Se, ≥99.5%), concentrated hydrochloric acid (HCl), and hydrazine hydrate (N_2_H_4_·H_2_O, 35%) were used. All the chemicals were purchased from Alfa Aesar and were used without further purification.

### Synthesis of Nanoplatelets

Bi_2_Se_3_ (*sample BiSe*) nanoplatelets were prepared using the hydrothermal method, as described in our previous research.^36^ In short, the stoichiometric amounts of bismuth nitrate (1 mmol) and selenium (1.5 mmol) were dissolved in 20 mL of deionized water under vigorous stirring, followed by an addition of 11 M HCl (25 μL) and hydrazine (1.6 mL). The obtained gray slurry was sealed in a Teflon-lined autoclave and heated at 240 °C for 48 h. Afterward, the autoclave was allowed to cool naturally to room temperature, and the product was washed several times with deionized water.

The Cr-doped Bi_2_Se_3_ nanoparticles were prepared with the same hydrothermal method as BiSe, with the difference that Bi was partially substituted with Cr. The amount of added Cr nitrate (denoted as the nominal Cr concentration) was set to obtain the theoretical composition of Bi_2–*x*_Cr_*x*_Se_3_ (*x* = 0.04–2, which corresponds to 2–40 at. % of Cr). In short, Cr nitrate, Bi nitrate, and Se were dissolved in 20 mL of water under vigorous stirring in a stoichiometric ratio of (Bi+Cr):Se = 1 mmol:1.5 mmol. Afterward, HCl and hydrazine were added. The obtained gray slurry was sealed in a Teflon-lined autoclave and heated at 240 °C for 48 h. After the autoclave cooled naturally to room temperature, the product was washed several times with deionized water and dried. The samples with added nominal Cr concentration ranging from 2 to 36 at. % are denoted as sample *BiSe-Cr_X*, where X denotes the stoichiometric nominal added concentration of Cr in at. % to obtain Bi_2–*x*_Cr_*x*_Se_3_ composition. The sample in which all Bi was substituted with Cr (40 at. %) is denoted as sample *CrSe*.

### Characterization

The synthesized product was characterized by a Rigaku MiniFlex X-ray powder diffractometer with Cu Kα radiation (λ-1541 Å, 30 kV, 10 mA). Lattice constants are determined using Rietveld refinement by fitting the peaks corresponding to the rhombohedral structure of Bi_2_Se_3_. For TEM analysis, the sample was suspended in a mixture of water and ethanol and deposited on a copper grid-supported Lacey carbon grid. The TEM and HAADF-STEM analysis was performed using a field-emission electron microscope (JEOL JEM-2100f UHR) operating at 100 keV equipped with an Oxford X-Max 80T EDXS apparatus. Quantitative STEM-EDXS analysis was done on hexagonally shaped nanoplatelets that display the rhombohedral structure of Bi_2_Se_3_ according to the selected area electron diffraction (SAED) pattern. The spectrum was recorded in regions where no presence of a secondary phase was observed or attached to the nanoplatelet. For undoped and each Cr-doped sample, 5–10 nanoplatelets were analyzed, where at least 10 EDXS spectra were obtained for each nanoplate. The same procedure was used to analyze the secondary phase. For each sample, the elemental concentrations were determined by statistical analysis of the values extracted from multiple spectra, providing the average and using the standard deviation as associated uncertainty. The same approach was applied to the secondary phase. The solid solubility of Cr in Bi_2_Se_3_ nanoplatelets was determined as a weighted average of the measured Cr concentrations on the different samples, considered with the corresponding uncertainty. The light absorption properties were analyzed with a classical UV–vis spectroscopy. The UV–vis spectroscopy was performed by a modular Ocean HDX spectrophotometer using a quartz cuvette with a size of 1 × 1 × 3 cm. The spectra were recorded in a range of λ = 200–1100 nm with 9 ms acquisition time. The measurements were performed on suspensions with a concentration of 0.25 g/L. Before the analysis, samples were sonicated to break larger agglomerates and make the suspension stable for the time of measurements.

For CL microscopy and spectroscopy, the field-emission scanning electron microscopy (SEM) apparatus (JEOL, JSM 7100f TTLS) is equipped with a parabolic mirror that collects the light and directs it toward a spectrometer (MonoCL4, Gatan). The probe current used for CL acquisition was 2 nA, with an acceleration energy of 15 keV. CL measurements were carried out at room temperature. In this study, the CL apparatus has been used for either panchromatic imaging or point spectroscopy. In panchromatic imaging, all of the emitted light is collected and directed onto the detector (photomultiplier tube), with a sensitivity range of ≈250–800 nm. The collected CL intensity signal is acquired during the imaging and represented in an 8-bit grayscale map (1280 × 1024 pixels) in parallel with the secondary electron (SE) signal. In point spectroscopy, the beam is kept at a selected position on the sample, the CL emission is directed into the spectrometer, and an intensity vs wavelength spectrum is acquired. The spectrometer is equipped with a grating (1200 lines/mm, 500 nm blaze). The entrance and exit slits were set in order to have a spectral resolution (bandwidth) of 10 nm. Spectra are acquired with a step size of 5 nm and a dwell time of 2 s. Data are presented in the manuscript without system response correction and background subtraction. Before the CL analysis, a quick EDXS analysis was performed to confirm that analysis is done only in the BiSe-Cr phase. The CL analysis was performed on similar-sized hexagonal nanoparticles, ranging from 100 to 200 nm.

## Results and Discussion

Figure 1a shows the XRD patterns of BiSe-Cr samples with different at. % of added nominal Cr concentration, compared to the diffraction pattern of pure Bi_2_Se_3_ nanoparticles, synthesized under the same conditions and reference Bi_2_Se_3_ card (JCPDS 33-0214). All patterns display peaks that can be indexed according to the Bi_2_Se_3_ rhombohedral structure (space group: *R*3*m*), except for the CrSe sample. The diffraction pattern of this sample, in which all Bi was substituted with Cr, can be indexed according to the trigonal structure of the mineral nevskite (COD 9012066). The peak, positioned at 2θ = 19.8, that could be indexed according to the nevskite structure can also be observed in the BiSe-Cr_36 sample (marked with an asterisk in Figure 1a). The Rietveld refinement analysis reveals a reduction of the cell parameters in the rhombohedral structure for BiSe-Cr samples compared to the pure Bi_2_Se_3_ nanoparticles synthesized (Figure 1b). The most significant decrease occurs along the *c*-axis.

**Figure 1:**
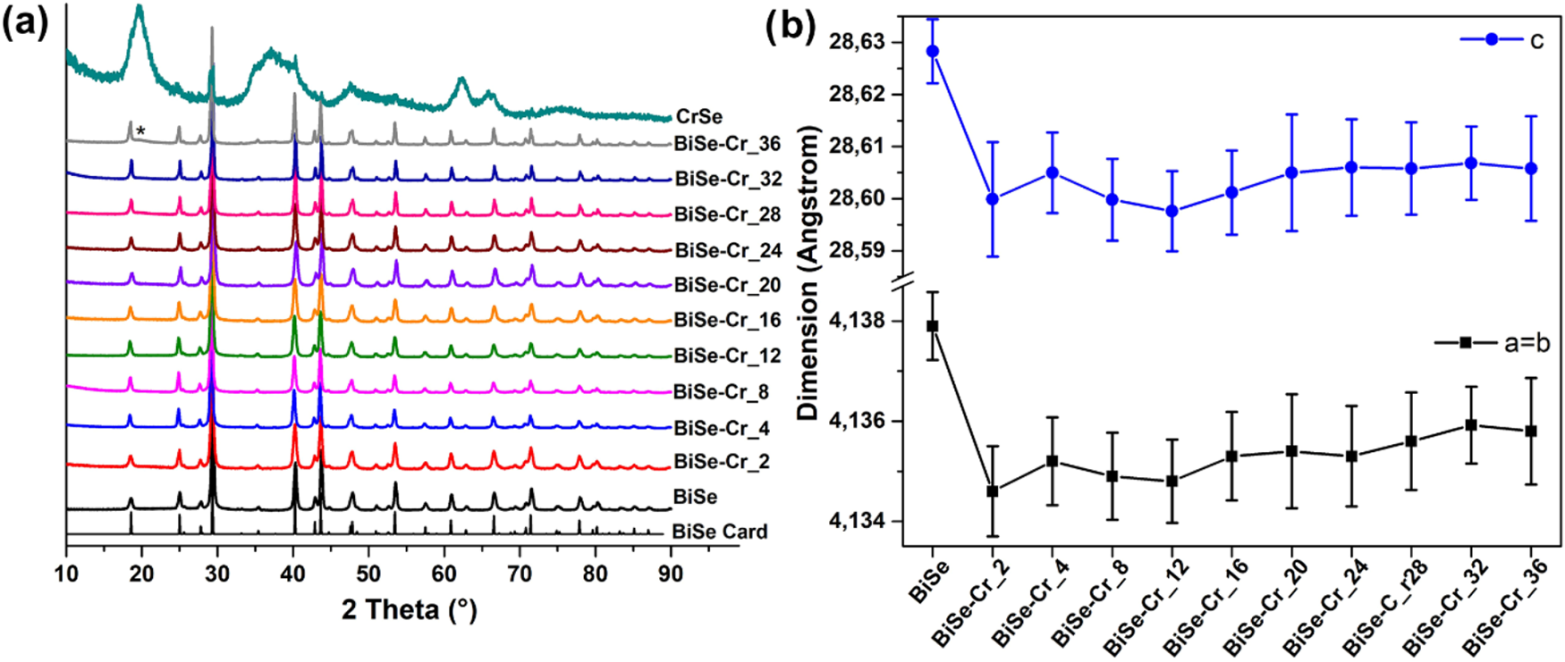
XRD patterns of Cr-doped Bi_2_Se_3_ nanoplatelets with different nominal Cr concentrations added (BiSe-Cr_X, where X is the nominal added Cr concentration in at. %), compared to the pristine Bi_2_Se_3_ (BiSe) sample and the reference card (JCPDS 33-0214) (a) as well as changes in lattice parameters calculated from Rietveld refinement (b). The presented XRD patterns are normalized to the highest intensity peak.

The reduction in the lattice parameter indicates substitutional incorporation of smaller Cr^3+^ ions (d_ionic_ = 0.755 Å) into the Bi^3+^ (d_ionic_ = 1.17 Å) sites. This reduction occurs as neighboring Se atoms move to accommodate the smaller radius of the Cr atom compared to that of Bi.^14,15^ However, after the initial decrease, the cell parameters do not decrease further despite increasing the nominal concentration of added Cr^3+^ ions in the reaction system. This indicates that we reached a threshold concentration, i.e., the solid solubility level for Cr^3+^ ions has been reached, beyond which Cr3+ ions can no longer substitute Bi^3+^ ions and incorporate into the rhombohedral structure of Bi_2_Se_3_ nanoplatelets.

Figure 2a shows a representative TEM image of the *BiSe-Cr_2* sample, revealing the presence of two phases: (1) large hexagonal nanoplatelets with lateral sizes of ≈100–800 nm and (2) smaller discoidal nanoparticles (≤20 nm) assembled into agglomerates. Both phases are observed also for all other samples (excepted BiSe), regardless of the added nominal Cr concentration. Selected area electron diffraction (SAED) data recorded from both the hexagonal nanoplatelets and the nanodisc agglomerates confirm that both phases are highly crystalline. The diffraction pattern recorded from the hexagonal nanoplatelet (Figure 2a, bottom right inset) can be indexed to the rhombohedral Bi_2_Se_3_ structure along the ⟨001⟩ zone axis, whereas the pattern acquired from the nanodisc agglomerates (Figure 2a, upper left inset) corresponds to the trigonal nevskite structure. These results demonstrate the presence of a structurally distinct secondary phase that cannot be resolved by XRD, highlighting the importance of TEM for nanoscale phase identification.

**Figure 2:**
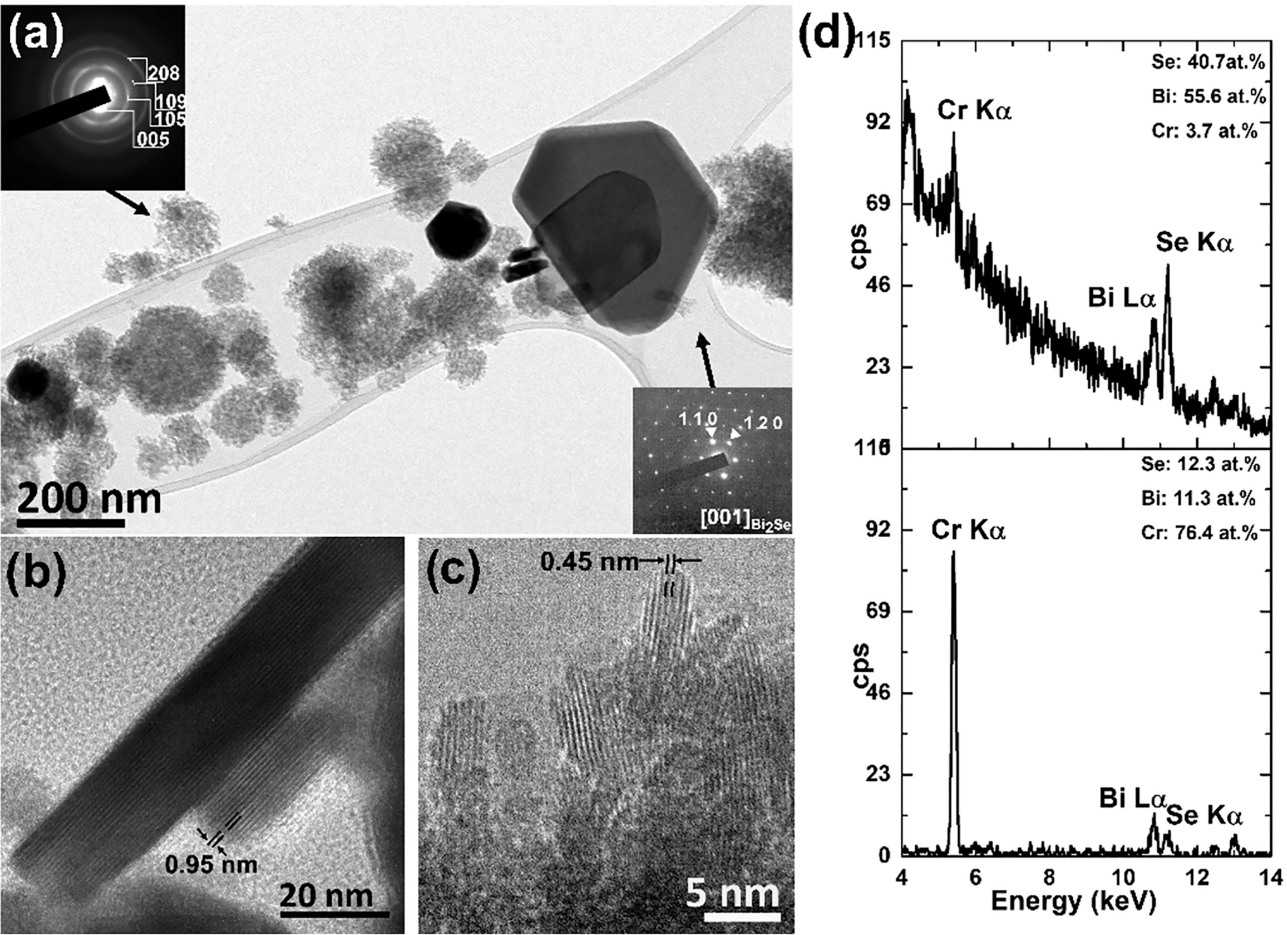
Representative TEM image of the BiSe-Cr_2 sample shows that the samples contain larger, hexagonally shaped nanoplatelets, which according to the electron diffraction (inserted on the bottom left corner) display the rhombohedral structure of Bi_2_Se_3_ and smaller, agglomerated discoid nanoparticles that according to the diffraction (inserted in the upper left corner) display the nevskite structure (a). HR-TEM images of the larger hexagonally shaped (b) and discoid (c) nanoparticles are shown, both oriented edge-on with its larger surfaces parallel to the beam and (d) EDXS recorded on large hexagonal nanoparticles (above) and on smaller discoid nanoparticles (below).

The structural differences between the two phases are most clearly resolved by HR-TEM when the anisotropic particles are oriented edge-on. The hexagonal nanoplatelet (Figure 2b) exhibits well-defined lattice fringes with a spacing of 0.95 nm, corresponding to the (003) planes of rhombohedral Bi_2_Se_3_. In contrast, the nanodiscs (Figure 2c) show lattice fringes with a spacing of 0.45 nm, consistent with the (005) planes of the trigonal nevskite structure.

Figure 2d presents STEM-EDXS spectra acquired from both nanoparticle types present in the sample BiSe-Cr_2. In both cases, the presence of the Cr Kα peak confirms chromium incorporation. The spectrum obtained from the hexagonal nanoplatelet (Figure 2d, top) shows a lower Cr signal relative to the Bi and Se signals, with an elemental ratio (Bi+Cr):Se of ≈ 2:3. This ratio corresponds to the expected Bi_2–_ *_x_* Cr*_x_* Se_3_ composition, identifying the hexagonal nanoplatelets as the Cr-doped Bi_2_Se_3_ phase in question.

Quantitative EDXS analysis on multiple hexagonally shaped nanoplatelets in the sample BiSe-Cr_2 shows that despite a nominal Cr addition of only 2 at. % and the presence of a secondary Cr-rich phase, the nanoplatelets contain 3.5 ± 1.5 at. % Cr. This deviation likely reflects the competition between primary and secondary phase formation during hydrothermal growth, resulting in local stoichiometric variations and Cr concentrations in the Bi_2_Se_3_ nanoplatelets that differ from the nominal composition. A fraction of unreacted precursor species can remain in solution and be removed during washing of the product.

Quantitative EDXS analysis of hexagonal nanoplatelets from BiSe-Cr_3–36 samples shows that as the nominal Cr concentration increases, the incorporated Cr content remains constant, an average value of 4.8 ± 0.4 at. % (Figure 3a). This reveals a solid solubility limit for Cr dopant incorporation in Bi_2_Se_3_ nanoplatelets prepared under hydrothermal synthesis conditions.

**Figure 3:**
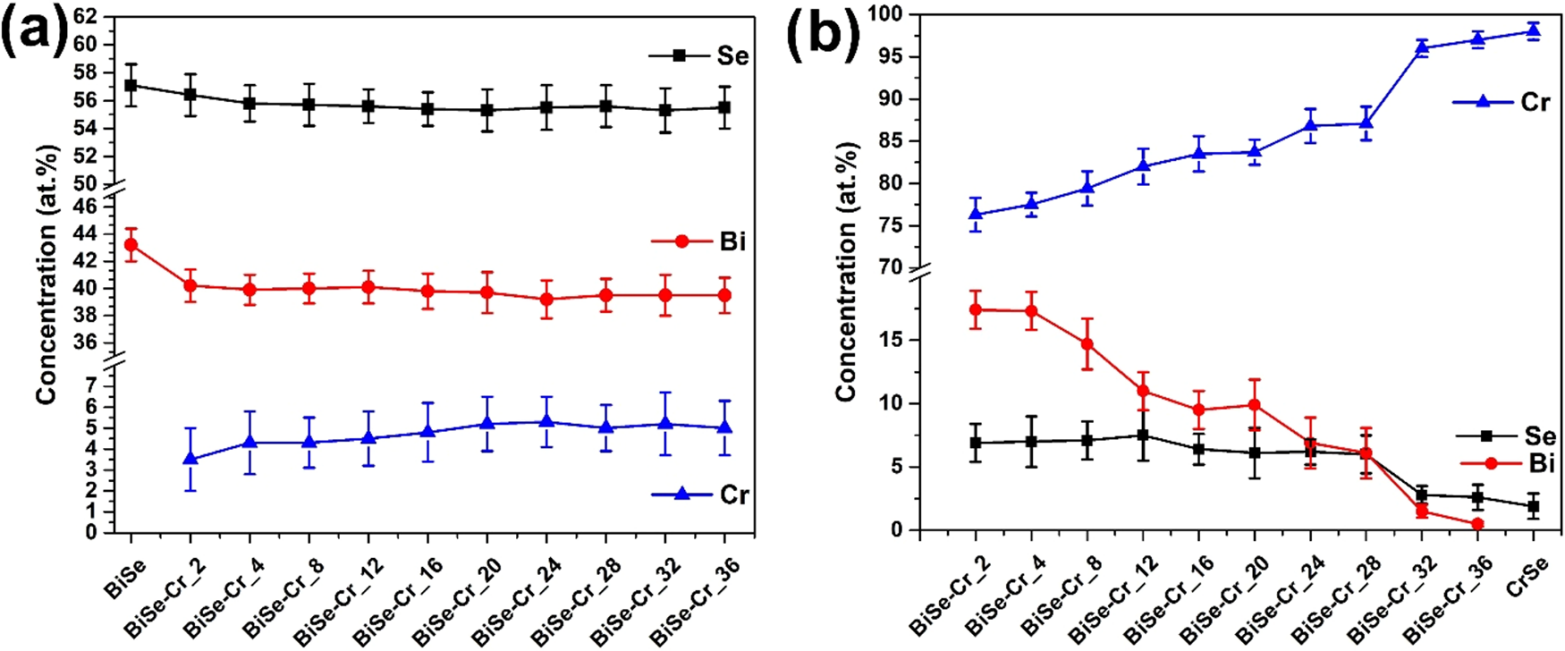
Bi, Se, and Cr concentration determined by EDXS for BiSe-Cr hexagonally shaped nanoplatelets (a) and secondary Cr-rich discoid nanoparticles (b).

The maximum Cr concentration obtained here is comparable to the maximum dopant levels previously reported for Bi_2_Se_3_ thin films prepared by MBE.^7,14–20^ Cr incorporation is accompanied by a corresponding decrease in Bi concentration, from 43.2 ± 1.2 at. % in pristine Bi_2_Se_3_ nanoplatelets to an average of 39.7 ± 1.5 at. % in Cr-doped samples, independent of the nominal Cr addition. In contrast, the Se concentration remains nearly unchanged at approximately 57.1 ± 1.5 at. %. These results confirm that Cr is incorporated into the Bi_2_Se_3_ lattice as Cr^3+^, substituting for Bi^3+^ ions.

In contrast, the spectrum acquired from the nanodiscs (Figure 2d, bottom) exhibits a much stronger Cr signal relative to that of Bi and Se, indicating a Cr-rich secondary phase. Compared to the Cr-doped Bi_2_Se_3_ nanoplatelets, where the Cr concentration is limited by the solid solubility level, this phase shows a clear compositional evolution, with Cr concentrations increasing from 75 to 95 at. % as the nominal Cr addition increases (Figure 3b). Despite these compositional variations, SAED patterns and lattice spacing measurements observed on the secondary phase confirm that this phase retains a consistent nevskite-type crystal structure.

Figure 4 shows the UV–vis absorption spectra of BiSe-Cr and CrSe samples, compared with pristine Bi_2_Se_3_ nanoplatelets. Samples BiSe-Cr_2–16 exhibit absorption behavior with spectral features similar to undoped Bi_2_Se_3_ across the entire spectral range (Figure 4a).^32,36,38^ They display absorption peaks in the 200–350 nm spectral region, which can be attributed to an optical response arising from bulk interband electronic transitions. At longer wavelengths (≈340–1100 nm), a broad absorption band is observed, which can be ascribed to interband and intraband transitions involving TSSs.^28^ Notably, the samples BiSe-Cr_2–8 exhibit enhanced absolute absorption compared to pristine Bi_2_Se_3_ over the whole measured region. This behavior can be attributed to the incorporation of Cr^3+^ ions, which introduce additional electronic states and optical transitions. Enhanced absorption has also been reported for Cr-doped oxide semiconductors such as TiO_2_ and SnO_2_, where Cr 3d states increase optical absorption.^39–43^ Despite the different crystal and electronic structure of Bi_2_Se_3_, the increased absorption observed here can also be attributed to Cr-induced optical transitions.

**Figure 4:**
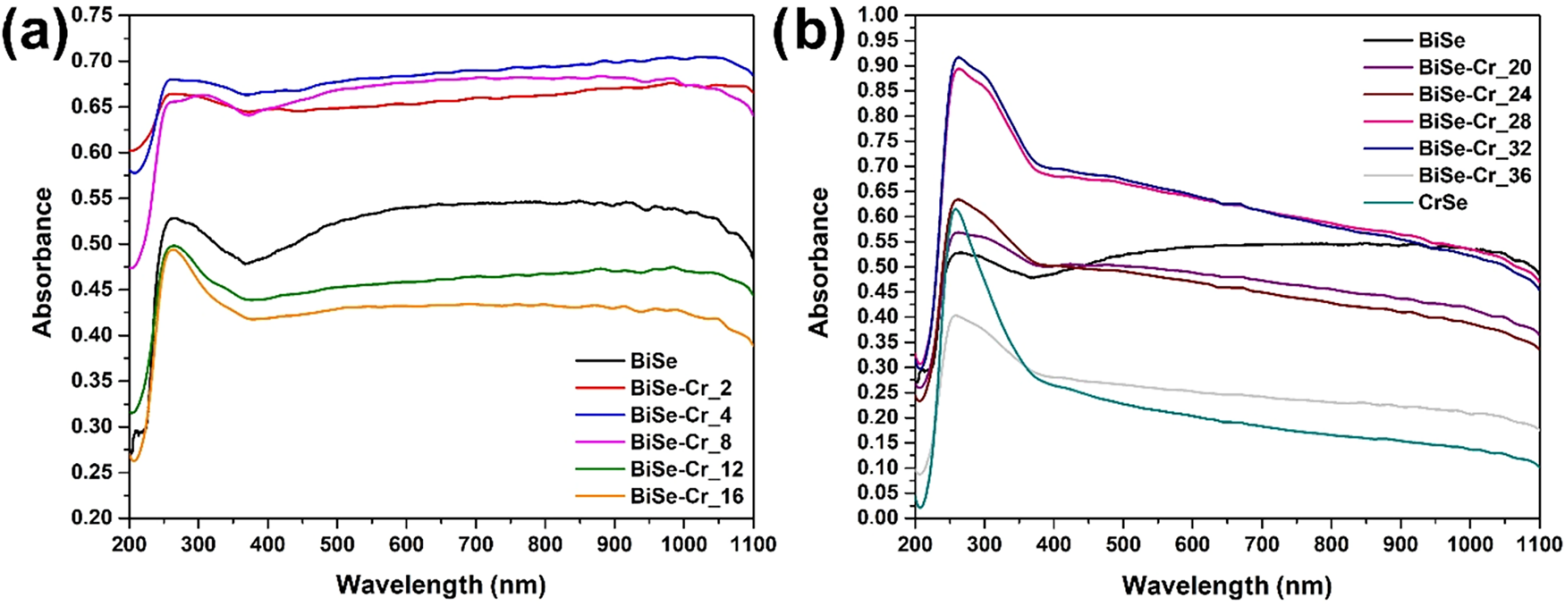
UV–vis absorption spectra of BiSe_Cr and CrSe samples compared with those of pure Bi_2_Se_3_ nanoplatelets (BiSe sample). For clarity, the results are divided into two parts according to the similarities in the line shapes of the absorption spectra of the pure Bi_2_Se_3_ phase (a) and CrSe phase (b).

Since EDXS analysis shows that increasing the nominal Cr precursor concentration does not increase the amount of Cr incorporated into the Bi_2_Se_3_ lattice, all Cr-doped samples contain Cr at the solid solubility limit, and therefore, it is expected that they would exhibit similarly enhanced UV–vis absorption compared to undoped nanoparticles. However, the BiSe–Cr_12 and BiSe–Cr_16 samples show a decrease in absolute absorption across the entire spectral range, even below that of the undoped nanoparticles. This behavior can be ascribed to an increasing fraction of the Cr-rich secondary phase. Consequently, its optical response contributes more strongly to the overall absorption, consistent with the Beer–Lambert law.

For samples containing >16 at. % of added nominal Cr concentration (Figure 4b), a pronounced change in the absorption profile is observed. While the absorption in the 200–350 nm region remains similar to that of pristine Bi_2_Se_3_ and BiSe–Cr_20–16 samples, the absorption at longer wavelengths (350–1100 nm) decreases monotonically without any distinct absorption peaks. Consequently, the spectra resemble those of the CrSe sample. Since EDXS analysis shows that the Cr dopant concentration in Bi_2_Se_3_ nanoplatelets remains constant with increasing nominal Cr addition and given the strong similarity of the BiSe–Cr20–36 spectra to CrSe, this behavior can be attributed to the increasing contribution of the Cr-rich secondary phase, whose optical response dominates in this spectral region.

UV–vis spectroscopy provides information on the average optical response of the sample. Therefore, direct evaluation of the intrinsic optical properties of Cr-doped Bi_2_Se_3_ nanoplatelets is hindered by the presence of Cr-rich secondary phases. To overcome this limitation, CL spectroscopy coupled with SEM was employed, enabling optical characterization on individual Cr-doped nanoplatelets with nanometer-scale spatial resolution and direct correlation with the local composition via EDXS. As we have recently demonstrated for related Bi_2_Se_3_ systems, CL spectroscopy is a powerful tool for probing intrinsic optical modes in pristine Bi_2_Se_3_ nanoplatelets.^37^

The panchromatic CL image of Cr-doped hexagonal nanoplatelets from the sample BiSe-Cr_2 (Figure 5a) reveals the differences in the emission intensity across the nanoplatelet when the electron beam is positioned at different locations on the nanoplatelet, e.g., edge or center (marked with A and B on Figure 5a, respectively. The CL emission is significantly more intense when the electron beam is positioned at the edges and corners of the nanoplatelet, while signal from the central region remains weak. Corresponding CL spectra acquired at the edge/corner (position A in Figure 5a,b) display a broad emission band cantered at ≈450 nm (3.1 eV) (Figure 5b). In contrast, the spectrum recorded from the nanoplatelet center (position B in Figure 5b) closely resembles the CL signal of the Si substrate (position C), indicating the absence of detectable emission from the nanoplatelet center. EDXS analysis performed at the same locations where CL spectra were recorded (Figure 5c) confirms that the Cr concentration is identical within experimental uncertainty at both the edge and the center of the nanoplatelet, demonstrating that the observed variation in CL emission does not originate from compositional differences. Instead, the emission remains localized at the edges and corners, consistent with previous reports on pristine Bi_2_Se_3_, where such behavior was attributed to wedge Dyakonov modes arising from the intrinsic hyperbolic electromagnetic response.^37,44,45^ This direct correlation between CL and EDXS further confirms that Cr incorporation at this concentration does not alter the intrinsic CL response compared to the pristine Bi_2_Se_3_ nanoplatelets.^37^

**Figure 5:**
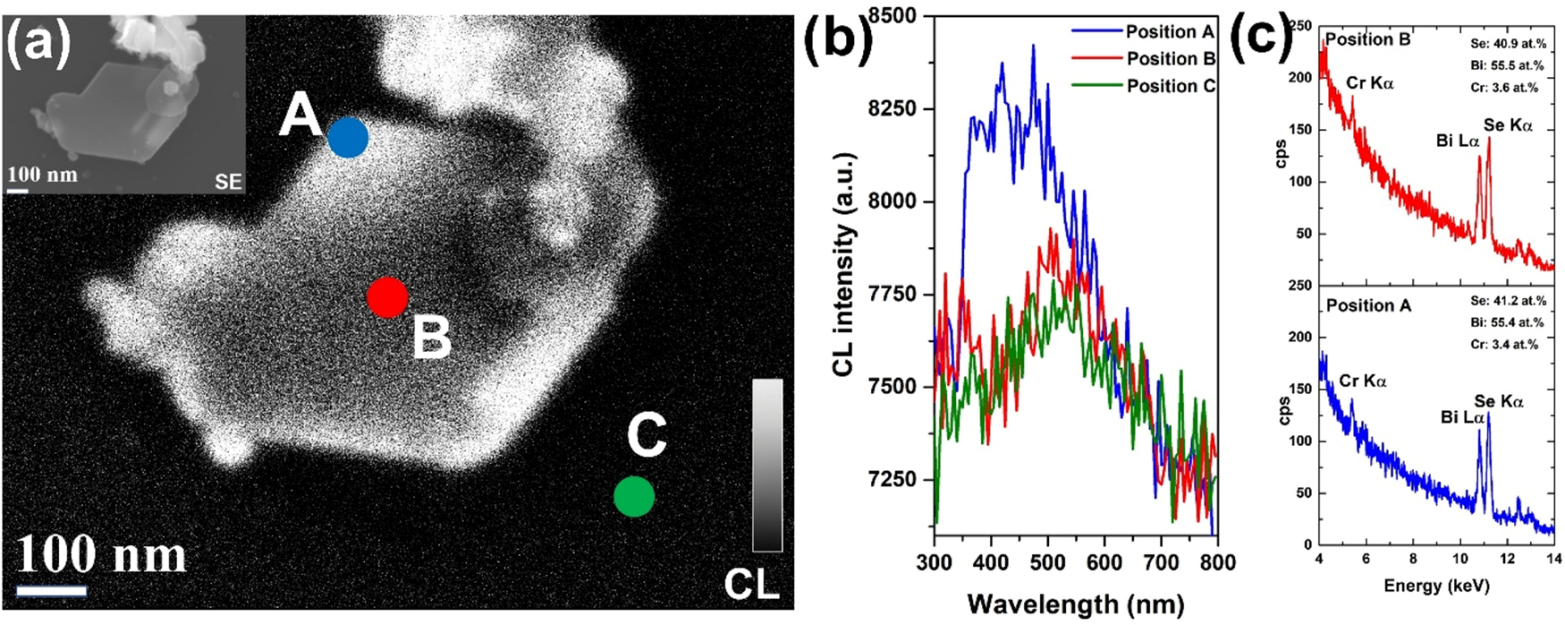
Panchromatic CL image (SE in the inset) with (a) corresponding CL spectra obtained by positioning the electron beam on a Cr-doped Bi_2_Se_3_ nanoplatelet as indicated by the colored dots in (b) and corresponding EDXS spectra obtained at the positions where CL spectroscopy was performed (c).

## Conclusions

Hydrothermal synthesis provides a viable route for Cr doping of Bi_2_Se_3_ nanoplatelets at the nanoscale. Substitutional incorporation of Cr into the Bi_2_Se_3_ lattice reaches 4.8 ± 0.4 at. % as determined by STEM-EDXS, already at the lowest Cr precursor concentration, and further increases in the nominal Cr content do not lead to higher incorporation, indicating a solid solubility limit of Cr in Bi_2_Se_3_ nanoplatelets. Excess of Cr results in the formation of a Cr-rich secondary phase. UV–vis absorption spectroscopy reveals enhanced optical absorption upon Cr incorporation; however, for samples with nominal Cr concentrations of ≥20 at. %, the optical response becomes dominated by the secondary Cr-rich phase. CL spectroscopy performed on individual Cr-doped nanoplatelets confirms preservation of the characteristic edge-localized emission with spectral features identical to those of pristine Bi_2_Se_3_,^37^ indicating that Cr incorporation does not alter the intrinsic emission modes. The findings of this study demonstrate that hydrothermal synthesis is a suitable, simple, and effective approach for producing magnetically doped Bi_2_Se_3_ nanoplatelets with dopant levels comparable to those achieved by MBE. As such, this synthesis represents an important step toward obtaining Cr-doped nanoparticles, which, based on the nature of the Cr^3+^ ion, may exhibit magnetic properties and could serve as a platform for future investigations of the interplay between magnetism and topology at the nanoscale.
